# Changing malaria fever test positivity among paediatric admissions to Tororo district hospital, Uganda 2012–2019

**DOI:** 10.1186/s12936-020-03490-4

**Published:** 2020-11-19

**Authors:** Arthur Mpimbaza, Asadu Sserwanga, Damian Rutazaana, James Kapisi, Richard Walemwa, Laurissa Suiyanka, David Kyalo, Moses Kamya, Jimmy Opigo, Robert W. Snow

**Affiliations:** 1 Child Health and Development Centre, Makerere University, College of Health Sciences, Kampala, Uganda; 2 Infectious Diseases Research Collaboration, Kampala, Uganda; 3 National Malaria Control Division, Ministry of Health, Kampala, Uganda; 4 Department of Prevention, Care and Treatment, Infectious Diseases Institute, Kampala, Uganda; 5 Population Health Unit, Kenya Medical Research Institute/Wellcome Trust Research Programme, Nairobi, Kenya; 6 Centre for Tropical Medicine and Global Health, Nuffield Department of Medicine, University of Oxford, Oxford, UK

**Keywords:** Changing, Test, Positivity, Paediatric admissions, Uganda

## Abstract

**Background:**

The World Health Organization (WHO) promotes long-lasting insecticidal nets (LLIN) and indoor residual house-spraying (IRS) for malaria control in endemic countries. However, long-term impact data of vector control interventions is rarely measured empirically.

**Methods:**

Surveillance data was collected from paediatric admissions at Tororo district hospital for the period January 2012 to December 2019, during which LLIN and IRS campaigns were implemented in the district. Malaria test positivity rate (TPR) among febrile admissions aged 1 month to 14 years was aggregated at baseline and three intervention periods (first LLIN campaign; Bendiocarb IRS; and Actellic IRS + second LLIN campaign) and compared using before-and-after analysis. Interrupted time-series analysis (ITSA) was used to determine the effect of IRS (Bendiocarb + Actellic) with the second LLIN campaign on monthly TPR compared to the combined baseline and first LLIN campaign periods controlling for age, rainfall, type of malaria test performed. The mean and median ages were examined between intervention intervals and as trend since January 2012.

**Results:**

Among 28,049 febrile admissions between January 2012 and December 2019, TPR decreased from 60% at baseline (January 2012–October 2013) to 31% during the final period of Actellic IRS and LLIN (June 2016–December 2019). Comparing intervention intervals to the baseline TPR (60.3%), TPR was higher during the first LLIN period (67.3%, difference 7.0%; 95% CI 5.2%, 8.8%, p < 0.001), and lower during the Bendiocarb IRS (43.5%, difference − 16.8%; 95% CI − 18.7%, − 14.9%) and Actellic IRS (31.3%, difference − 29.0%; 95% CI − 30.3%, − 27.6%, p < 0.001) periods. ITSA confirmed a significant decrease in the level and trend of TPR during the IRS (Bendicarb + Actellic) with the second LLIN period compared to the pre-IRS (baseline + first LLIN) period. The age of children with positive test results significantly increased with time from a mean of 24 months at baseline to 39 months during the final IRS and LLIN period.

**Conclusion:**

IRS can have a dramatic impact on hospital paediatric admissions harbouring malaria infection. The sustained expansion of effective vector control leads to an increase in the age of malaria positive febrile paediatric admissions. However, despite large reductions, malaria test-positive admissions continued to be concentrated in children aged under five years. Despite high coverage of IRS and LLIN, these vector control measures failed to interrupt transmission in Tororo district. Using simple, cost-effective hospital surveillance, it is possible to monitor the public health impacts of IRS in combination with LLIN.

## Background

Indoor Residual Spraying (IRS) has regained attention as a means to prevent malaria infection in high transmission areas of Africa when used in combination with insecticide-treated nets, largely as a result of funding made available by the US President’s Malaria initiative (PMI) [1]. Carbamates, organochlorines, organophosphates, pyrethroids, and more recently neonicotinoids, have been used in 23 countries in SSA since 1997, resulting in approximately 21 million people being protected by one form of IRS in 2017 [2].

In Uganda, between 1959–1964, IRS using dichloro-diphenyl-trichloro-ethane (DDT) with three rounds of mass drug administration (MDA) with single doses of chloroquine-pyrimethamine was piloted in Kigezi and parts of Masaka districts [3]. During the first year of the operations parasite prevalence declined from 16.6 to 0.3% [4]. Between 1963 and 1964, a large-scale field trial of Malathion was carried out in Masaka district reducing vector densities to 0.0011 from 66 per house per day within a year [5]. Following on from these successes, the Uganda malaria pre-eradication programme was established in 1964 as a joint Government of Uganda-WHO project [6]. However, the period between 1970 and 1990 was characterized by a general collapse of the country’s health system as a result of civil war and political unrest. During this period, IRS alongside other forms of malaria prevention were largely abandoned.

Lambda-cyhalothrin IRS was introduced in 2004 to tackle localized epidemics in Kabale, Kisoro and Kanungu districts. In 2005, Uganda became one of the first countries in sub-Saharan Africa to benefit from PMI funding, supporting a sustained IRS campaign in different parts of the country. From 2006, IRS with lambda-cyhalothrin was introduced to Kabale and Kanungu districts [7]. PMI provided their implementing partner over US$ 150 million between 2007–2012 to continue, and expand, IRS activities with lamda-cyhalothrin across districts in the north (Lamwo, Kitgum, Pader, Agogo, Kole, Oyam, Apac, Gulu, Amuru and Nwoya) and Kanungu [8, 9]. For a brief period in 2008, the Ministry of Health used DDT as part of IRS programs in Apac and Oyam districts. In Katakwi and Kumi, in the Teso region, between 2008–2009 lambda-cyhalothrin was used in combination with mass screening and treatment as a combined intervention aimed at district-wide elimination [10].

In 2009, both DDT and pyrethroid resistance were detected at IRS districts [11] and the IRS programs switched to spraying with carbamate (Bendiocarb) [12]. Epidemiological impacts were few, however, at Apac district a 45% reduction in community parasite prevalence in children under five years was attributed to IRS using DDT (2007–08) and Bendiocarb (2009–10) [12], and a 62% reduction in health service use for malaria morbidity was associated with Bendiocarb IRS [13].

Between April and October 2014, IRS was scaled down in the northern districts (Acholi sub-region) and switched to seven mid-northern districts (Lango sub-region: Alebtong, Amolator, Dokolo, Kabaremaido, Lira, Otuke) and eight eastern districts (Serere, Pallisa, Kibuku, Budaka, Namutumba, Butaleja, Bugiri, Tororo) initially using Bendiocarb and later switching to Actellic 300 CS (organophosphate pirimiphos-methyl). Cessation of IRS in the Northern Districts resulted in large-scale epidemics from April 2015 through to 2016 affecting over 1 million people [14]. At Apac, 18 months following cessation of IRS, malaria morbidity had returned to pre-IRS baseline levels [15].

Unlike the introduction of IRS during the late 1950s, the rapid expansion of IRS in Uganda from 2007 was not accompanied by a comprehensive, longitudinal epidemiological impact analysis plan across all districts where IRS was implemented [6]. There has been one notable exception involving detailed parasitological, clinical and entomological studies at Nagongera sub-County in Tororo district since 2007 [16–22]. These studies provided conclusive evidence that with > 80% LLIN coverage and four rounds of IRS (coverage ranging between 85–100%), the incidence of malaria among children was reduced by 92% and the prevalence of infection by 93% [20, 22]. No studies have been undertaken to assess the impact of IRS on child survival or hospitalization in Tororo District. Here, temporal monthly data have been assembled from a hospital surveillance system at Tororo district hospital to examine the impact of vector control approaches on paediatric malaria infections admissions and their age patterns since 2012 to 2019.

## Methods

### Study area and malaria control activities

**Fig. 1:**
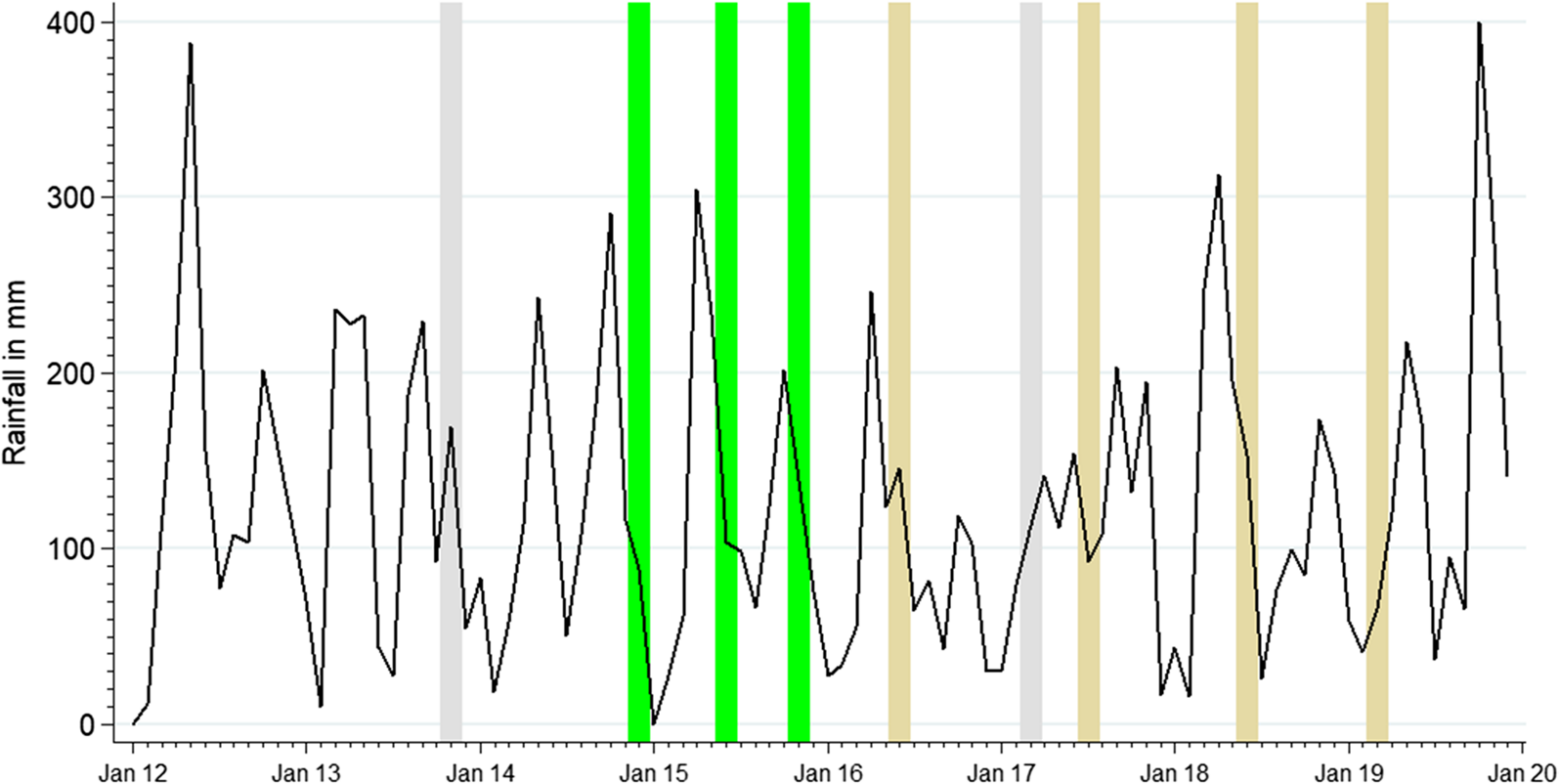
Rainfall pattern (black line) and distribution of vector control interventions in Tororo district 2012 to 2019. Vertical grey bars represent two rounds of mass distribution of long lasting insecticidal nets. The green bars show the timing of 3 rounds of IRS with Bendiocarb, brown bars 4 rounds of Actellic

Tororo District is in Bukedi sub-region, a historically highly malaria endemic, rural area of eastern Uganda bordering Kenya. The 2014 national census put the population of Tororo District at 517,082 [23]. The area supported an estimated entomological inoculation rate of 310 infective bites per person per year in 2011–12, and children aged under 10 years could expect between 2–3 clinical events per year with prevalence of community infections in this age group > 35% [24]. Malaria transmission is perennial, however there are two annual peaks coincidental with the two rainy seasons (March–May and August–October) (Fig. 1). Prior to 2014, malaria transmission was maintained predominantly by *Anopheles gambiae *sensu stricto (*s.s*.) (82%) and *Anopheles arabiensis* (19%) [25]. Post-2014 following IRS, *An. arabiensis* (99.5%) became the dominant vector [19].

The public health care system in Tororo district covers four tiers of out-patient services including 61 level II and III health centres (HCs) providing the most basic services and three level IV HCs providing more advanced levels of care in the form of some in-patient care. There are two hospitals providing general in-patient care, including the main Tororo district hospital with 52 in-patient paediatric beds. The fee-paying St Antony’s mission hospital, also located in Tororo town, admits few patients, and does not serve as a major competitor to the government hospital. The nearest Regional Referral Hospital is at Mbale, 46 km south of the district. Since 2006, artemether-lumefantrine (AL) has been used as the first-line treatment for uncomplicated malaria in Uganda. In 2011, artesunate-amodiaquine was introduced as an alternative first-line treatment to AL [26]. Since 2011, the national standard treatment guidelines have promoted the parasitological diagnosis of malaria at all levels of the health sector, through either rapid diagnostic tests (RDTs) or microscopy [26].

The Government of Uganda has maintained routine distributions of long-lasting insecticide-treated nets (LLIN) since 2007 through antenatal clinics [27]. Mass campaigns of household distributions of LLIN were undertaken in May 2010, November 2013, and June 2017 (Fig. 1). In August 2011, it was estimated that 42% of the total population were protected by a LLIN [28]; in December 2014, 65% of the total population were reported sleeping under a LLIN the night before the survey [29]; declining to 46% in October 2016 [30]; and rising again to 69% in January 2019 of the sampled population of Tororo [31].

IRS began in Tororo district in December 2014-February 2015 and was repeated in June-July 2015, and November–December 2015 using Bendiocarb (Fig. 1). As a result of resistance to carbamates, annual IRS was switched to the long-lasting organophosphate pirimiphos-methyl (Actellic) from June 2016 [19, 32] (Fig. 1); with studies showing that the main malaria vector*, An. gambiae *sensu lato (*s.l*), has been 100% susceptible to Actellic [33]. In November 2019, 99% of school children included in a malaria survey in the district reported living in houses that had been sprayed in the last 12 months [34].

### Hospital surveillance

The present study was undertaken using secondary, retrospective analysis of surveillance data at Tororo district hospital. Data were collected as part of an inpatient surveillance program that was initially set up for surveillance of inpatient malaria, but later expanded to surveillance of Acute Febrile Illness (AFI). Details of the programme are described elsewhere [34–36]. In brief, a structured Medical Record Form (MRF) was used to capture standardized residential, medical history, clinical examination, and laboratory results on all admissions. Since its inception the surveillance program has supported malaria diagnostic testing (microscopy or rapid diagnostic testing) and emphasized good medical record keeping for all hospitalized children.

Analysis was limited to children resident in Tororo district at the time of hospitalization between January 2012 and December 2019. To determine the physical location of individual households, relevant address information (village, parish, sub-county, and district) recorded on patient MRFs was used to map residences of all admissions to the hospital. Where insufficient information was available on the MRF, missing address details were crosschecked against original hard copy admission registers. If residential information was still missing, admissions were excluded from analysis. Age was calculated in months from reported/documented date of birth and admission date. Neonates were excluded from the analysis and therefore analysis was restricted to children aged 1 month to 14 years.

### Data entry and analysis

Between 2012 and 2015, complete MRFs were entered into an Access database (Microsoft Corporation, Redmond, WA, USA) and transmitted to a central database located in Kampala, Uganda on a monthly basis. From 2016, the data management system was upgraded to a web-based data management system hosted on the District Health Information System (DHIS-2), allowing for real time data entry. Data were analysed using STATA (version 14; STATA Corp., College Station, TX, USA). Monthly malaria test positivity rate (TPR) measured over time formed the basis of the primary analysis and was defined as the number of hospitalized children with a fever (temperature ≥ 37.5 °C and/or a history of fever in the presenting illness) and a positive malaria test result (RDT or microscopy) among all hospitalized febrile children with a malaria test result. The period of observation ranged from January 2012 to December 2019 and was divided into four time-periods. First, the baseline period, starting January 2012 to October 2013, characterized by routine malaria control interventions. Second, from November 2013 to November 2014, representing the period immediately after the first universal LLIN campaign and before the start of IRS in the district. Third, from December 2014 to May 2016, representing a period of three rounds of IRS with bendiocarb, implemented at six monthly intervals. Fourth, from June 2016 to December 2019, representing four rounds of IRS with Actellic, implemented annually, with a second universal LLIN distribution campaign in the district. Two analytical approaches were used to determine the impact of interventions on TPR during the observation period; before-and-after analysis and interrupted time series analysis (ITSA).

For before-and-after analysis, the impact of interventions on TPR was assessed by calculating the absolute difference between the aggregated baseline TPR (before) with the three intervention intervals (after): (1) first universal LLIN campaign, (2) IRS with Bendiocarb, and (3) IRS with Actellic and second LLIN campaign. The chi square test was used to assess if differences in TPR were statistically significant. The significance level, alpha, was set at 0.05. The 95% CI was determined based on the absolute difference in TPRs plus or minus the margin of error; a product of the standard deviation of the difference between periods and the critical value (Z = 1.960). To explore more immediate effects of interventions on TPR, data were also repeated limited to children aged 1–11 months.

ITSA [37–40] was used to assess the effect of IRS on TPR during the observation period January 2012 to December 2019, based on monthly changes in TPR as opposed to aggregated period TPR. The aim was to examine the impact of IRS on monthly-adjusted TPR values during sustained periods of LLIN distribution and use. Therefore, the two observation periods were divided into (a) the first, pre-IRS intervention period starting January 2012 to December 2014 combining the baseline and first LLIN campaign period; and (b) the post-IRS intervention period starting January 2015 to December 2019 combining IRS (Bendiocarb and Actellic) with the second LLIN campaign periods. The end month of the first Bendiocarb IRS campaign was used to reflect the time when implementation of the intervention was completed. To model the effect of IRS on TPR, ordinary least squares regression was used with Newey West standard errors to handle autocorrelation with a 1-month lag. To adjust for possible confounding, models were adjusted for estimates of monthly rainfall in millimetres (recorded at Tororo town) with a 1-month lag, mean age (months) and type of malaria test done (the percentage of all tests done that were RDT). The model estimated the immediate effect of Bendiocarb on TPR, the monthly rate of change in TPR during the pre-intervention period, and the difference between the pre- and post-intervention trends.

Age (months) of hospitalized children with malaria was summarized as median (inter quartile range) and mean (± standard deviation) for the baseline period and different intervention periods stratified depending the malaria test result. A Kruskall-Wallis test was used to test for differences between age during different intervals. The linear temporal change in mean age among test positive hospitalized were estimated using linear regression adjusted for 1 month lagged rainfall and type of test done.

## Results

### Characteristics of the study population

Across the 96 months of observation, January 2012 to December 2019, 33,643 admissions aged ≥ 1 month to 14 years were recorded at Tororo District hospital. 1553 (4.6%) admissions were residents of neighbouring districts or Kenya, 194 (0.6%) children had insufficient address information to define district of residence. Among the 31,896 hospitalized children aged 1 month to 14 years resident in Tororo District, 28,049 (90%) had a fever defined as a temperature ≥ 0.5 °C or reported history of fever, and 25,884 (93%) of these children had a malaria test performed at, or during, admission (Additional file 1: Table S1). Overall, among children with fever the TPR was 48.3%, range 19.1% to 68.6% over the eight years of observation. TPR decreased from 58.5% in 2012 to 28.3% in 2019, and in children aged 1 to 11 months from 53.6% to 15.4%. The mean and median ages of children with a positive malaria test results increased over time (Additional file 1: Table S1).

### Before and after comparison of TPRs

Compared to the baseline TPR (60.3%), TPR was higher during the first LLIN campaign period (67.3%, difference 7.0%; 95% CI 5.2%, 8.8%, p < 0.001), but was lower during the Bendiocarb IRS (43.5%, difference − 16.8%; 95% CI − 18.7%, − 14.9%, p < 0.001) and Actellic IRS (31.3%, difference − 29.0%; 95% CI − 30.3%, − 27.6%, p < 0.001) (Table 1). Compared to the first LLIN campaign period TPR (67.3%), TPR decreased during the Bendiocarb IRS period (43.5%, difference − 23.8%; 95% CI − 26.0%, − 21.6%, p < 0.001). Finally, compared to the first LLIN campaign and Bendiocarb IRS period, TPR was lower during the Actellic IRS period combined with the second LLIN campaign (31.3%, difference − 36.0%; 95% CI − 37.7%, − 34.2%, p < 0.001; difference − 12.2%; 95% CI − 14.0%, − 10.3%, p < 0.001, respectively) (Table 1). A similar pattern was observed when analysis was limited to children aged 1–11 months, however the difference following the switch from Bendiocarb to Actellic IRS showed a more dramatic decline (34.3% vs. 16.6% difference − 17.7%; 95% CI − 21.0%, − 14.4%, p < 0.001) (Table 1).

**Table 1:** Test positivity rate among hospitalized children at Tororo district hospital during different time periods: 2012 to 2019

Variable	Unit	Period			
		Baseline	1st mass LLIN campaign	IRS campaign with Bendiocarb	IRS with Actellic and 2nd mass LLIN campaign
		January 2012 To October 2013	November 2013 To November 2014	December 2014 to May 2016	June 2016 to December 2019
Total admissions	N	9956	4712	4784	12444
Admissions with fever	n/N	9293/9916	4154/4422	3897/4683	10705/12142
	%	93.7%	93.9%	83.2%	88.1%
Admissions with fever tested for malaria	n/N	9073/9239	3771/4154	3599/3897	9441/10705
	%	97.6%	90.8%	92.3%	88.1%
TPR among tested with fever	n/N	5469/9073	2537/3771	1565/3599	2955/9441
	%	60.3%	67.3%	43.5%	31.3%
TPR among infants tested with fever	n/N	1614/2932	630/1090	341/994	369/2226
	%	55.0%	57.8%	34.3%	16.6%
Fever positives aged 1 to 11 months	n/N	1614/5469	630/2537	341/1565	369/2955
	%	29.5%	24.8%	21.7%	12.5%
Fever positives aged 5 to 14 years	n/N	469/5469	261/2537	220/1565	670/2955
	%	8.6%	10.3%	14.0%	22.7%
Age (months) for fever positives	Median (IQR)	17 (10, 29)	18 (12, 36)	22 (12, 36)	30 (17, 48)
	Mean (SD)	23.7 (21.6)	26.7 (24.7)	29.6 (25.3)	39.0 (30.9)

### Interrupted time series analysis: effect of IRS on monthly changes in TPR

**Fig. 2:**
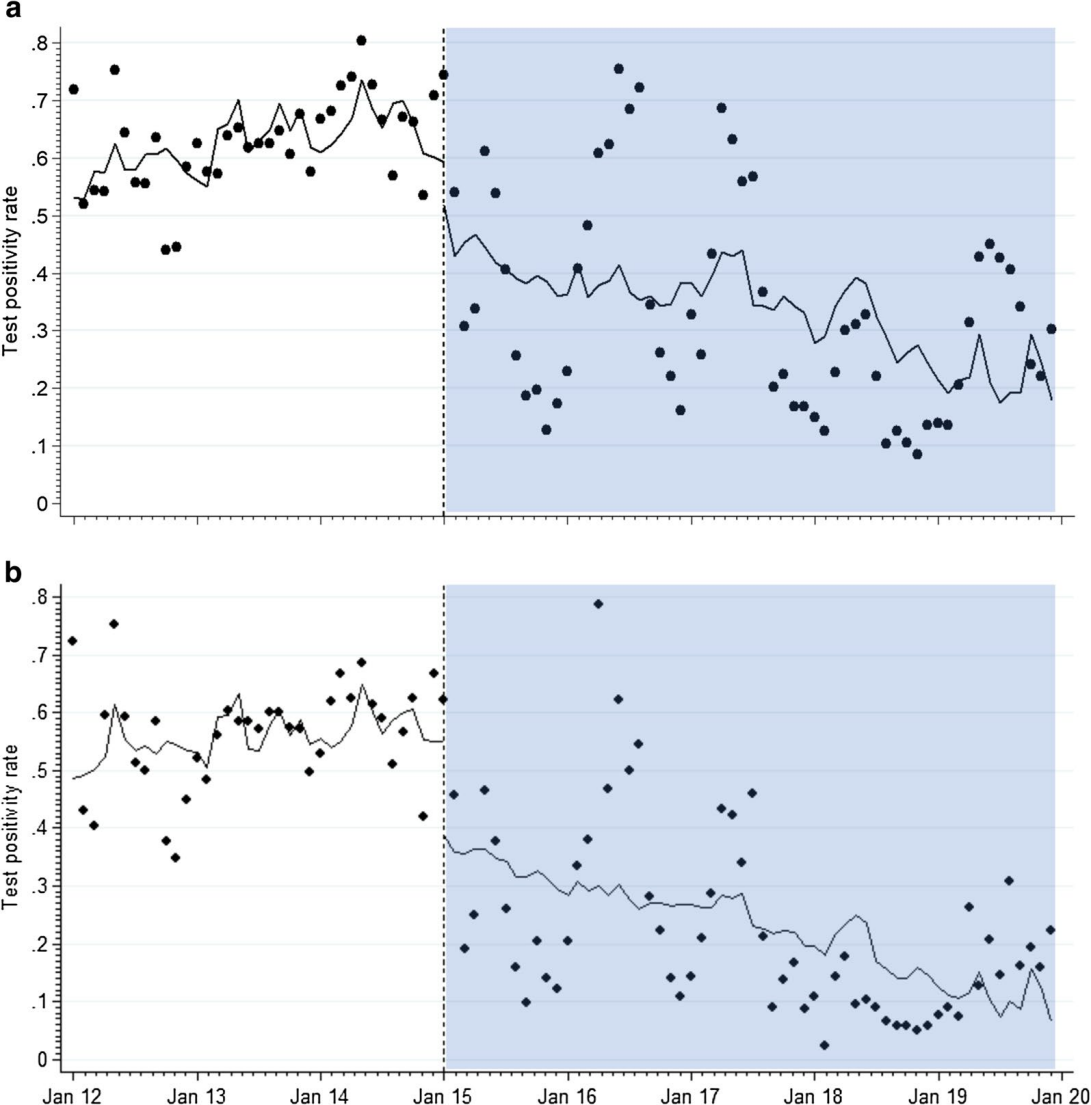
**a** Interrupted time series analysis of TPR with Newey-West standard errors and one lag for test positivity rates in relation to initiation of IRS with Bendiocarb (vertical line) in January 2015 among all hospitalized children. The shaded area represents the post-IRS intervention period; IRS (Bendiocarb and Actellic) combined with the second LLIN campaign periods. The pre-IRS intervention period (January 2012 to December 2014) is unshaded (white); the baseline period combined with first LLIN period. **b** Same as (**a**), but limited to infants aged 1 to 11 months

During the pre-IRS period; combined baseline and first LLIN period (January 2012 to December 2014), monthly rainfall, age and test type adjusted TPRs among children aged 1 month–14 years did not change significantly (Fig. 2a). Immediately after the first Bendiocarb IRS campaign, there was a significant drop in the adjusted TPR by − 14.8% (95% CI − 25.6%, − 2.3%, p = 0.022) the month after, followed by a decline in the adjusted TPR at a monthly rate of − 0.6% (95% CI − 0.9%, 0.2%, p = 0.005) during the combined IRS (Bendiocarb + Actellic) and the second LLIN campaign periods. This trend in the monthly decline of TPR post-IRS was significantly different from the monthly TPR trends during the pre-IRS period (baseline + first LLIN campaign) (difference in trends − 0.6%, 95% CI − 1.0%, − 0.1%, p = 0.024) (Fig. 2a). Similar trends post-IRS, without adjustment for age, were noted when analysis was restricted to children aged 1 to 11 months (p = 0.005) (Fig. 2b).

### Changes in age of test-positive admissions

**Fig. 3:**
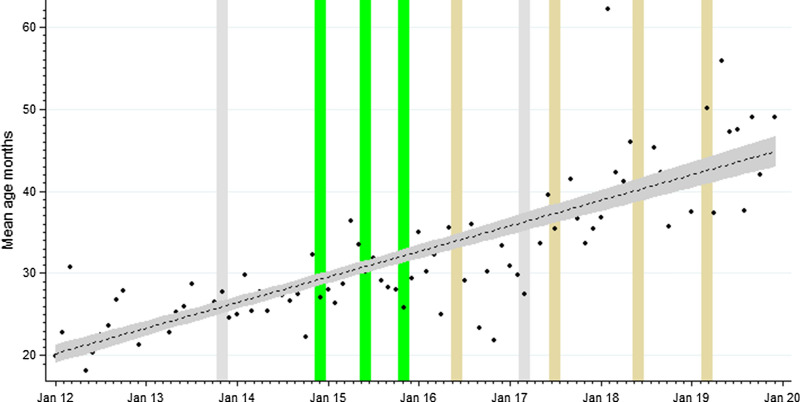
Monthly trends in the mean age (months) of hospitalized children with positive malaria test result at Tororo district hospital January 2012 to December 2019. A linear best fit line shown as a dashed line. Credible confidence represented as 95% CI (area shaded grey). Interventions represents as shown in Fig. 1

The monthly mean age of febrile paediatric admissions with a positive malaria test result steadily increased through the baseline and intervention periods (p = 0.001); lowest during the baseline period; median 17 months (IQR 10, 29), mean 23.7 months (SD 21.6) and highest during the last intervention period of IRS with Actellic; median 30 months (IQR 17, 48), mean 30 months (SD 30.9). The regression analysis, adjusting for lagged rainfall and test type showed a significant monthly increase from January 2012 to December 2019 in the mean age of febrile test-positive admissions (Fig. 3: coefficient 0.265, p = 0.001).

## Discussion

The analysis of fever malaria test-positivity rates at Tororo District Hospital from January 2012 to December 2019 highlights significant declines associated with the introduction of IRS in the district (Table 1; Fig. 2). Prior to the introduction of Bendiocarb IRS in December 2014, during a period when the only form of vector control was routine or mass delivery of LLIN, the average TPR among febrile hospital admissions was 63% (Table 1). However, TPR declined during the periods of IRS use to 43% (Bendiocarb) and 31% (Actellic) (Table 1). Interrupted time-series analysis, controlling for age, rainfall patterns and type of test, shows that compared to the pre-IRS period, IRS (Bendiocarb and Actellic) with the second LLIN campaign period resulted in significant overall monthly reductions in TPR (Fig. 2). Importantly, in this setting of historically high transmission the results demonstrate the combined added value of IRS and LLIN, compared to LLIN alone, findings supported across different settings in Africa [18, 41, 42].

The findings presented here correspond with longitudinal studies of community infection prevalence 2007–2012 (Pre-IRS) and 2014–2017 (Post-IRS, without chemoprophylaxis) among a cohort of children aged 6–30 months in Nagongera sub-county, Tororo [20] and the dramatic reduction in the incidence of clinical malaria in the same cohort [21, 32]. A comparison with the patterns of out-patient TPR at Nagongera Health Centre [18, 43], and the hospital TPR (Table 1) showed identical patterns of change.

Throughout the time-series the mean age of paediatric febrile admission malaria test positives increased from 24 months in 2012–2013 to 46 months in 2019 (Fig. 3; Table 1; SI Table 1). Among test-positives during the baseline period (January 2012–October 2013), 29.5% were aged 1–11 months and 8.6% were aged 5–14 years, conversely during the last period of Actellic IRS (June 2016–December 2019), 12.5% were aged 1–11 months and 22.7% were 5–14 years (Table 1). Changing age-patterns of TPR have also been shown among febrile, paediatric out-patient attendees associated with sustained vector control elsewhere in Uganda [43] and Mozambique [44]. The majority of febrile admissions associated with malaria infection continue to be aged < 5 years (87%), however the changing age-pattern among young children suggests a possible impact of sustained, high coverage vector control on the acquisition of immunity among childhood populations [45].

Despite the entomological inoculation rate in Nagongera sub-county declining from 129 infective bites per person per year in 2013 to an apparent zero in 2017 [19], findings from this study suggest that transmission has clearly not been interrupted in the district. During the last period of Actellic IRS (June 2016 to December 2019), 31% of children with fever and tested for malaria presented to the district hospital still harboured malaria infections (Table 1). Limiting the data to all admissions aged 1–11 months, reflecting more recent community transmission in 2019, 90/857 (10.5%) were either slide or RDT positive. Malaria therefore continues to be associated with a substantial proportion of hospitalizations in the district, despite high coverage of LLIN and IRS using a currently efficacious residual organophosphate. The experimental addition of monthly chemoprophylaxis in Nagongera appears to accelerate a reduction in transmission when used in combination with IRS [17, 20, 22]. Similar findings have been observed when IRS is combined with Mass Drug Administration (MDA) in Zambia [46] and Mozambique [47]. Combinations of vector and chemoprevention of parasite reservoirs are likely to accelerate reductions in transmission [48]. However, the complete interruption of transmission in Uganda is as challenging today as it was during early experimental studies of IRS and drug-based interventions during the 1960s [3–5]. Furthermore, monthly TPR values varied significantly during periods of IRS (Fig. 2), suggesting variations with time since spraying but importantly that any cessation of IRS in Tororo is likely to rapidly result in resurgence of infections among paediatric admissions. Uganda has ample evidence of the dramatic, immediate impact of removing intensive vector control though IRS on out-patient and in-patient hospital burdens [14, 15]. Relying only on moderate-to-high LLIN coverage alone will not mitigate this potential change in malaria burdens.

The WHO recommends that IRS or universal LLIN coverage should be selected as district-wide vector control, not both [49]. The available controlled trial evidence suggests little additional gains of combined LLIN and IRS as compared to the effective implementation of either approach independently [49]. However, in practice national malaria programmes, such as Uganda, continue with LLIN distribution in areas selected for IRS with the benefit of mitigating insecticide resistance [49]. The combination of IRS and LLINs had greater impact on TPR than LLIN used in isolation (Fig. 2; Table 1). This may have arisen because of LLIN and IRS campaigns used different classes of insecticides during a phase of emerging pyrethroid resistance [50, 51]; LLIN distributions have never achieved 100% coverage in Tororo [31], high bed net attrition rates [52], and those who own LLINs might not always use them [53]. Importantly, as shown in Uganda, sustaining IRS is heavily dependent on donor support, and when donors elect to change support for IRS, or the location, it is essential that these communities continue to be protected by LLIN.

The use of fever TPR has avoided the attribution of admissions to a specific admission diagnosis of malaria, in part because admissions have multiple causes, but largely because fever surveillance for infection is a much simpler, less ambiguous surveillance outcome. Unlike routine health information systems in Uganda which provide only crude age groupings above and below 5 years of age, it has been possible in the hospital analysis presented here to explore in more detail the precise age of test positives with time. However, long-term hospital surveillance suffers from several intrinsic problems that require contextualization, for example changing diagnostic protocols, access to diagnostics and patient access. Furthermore, TPR is less sensitive to measuring the true magnitude of the impact of interventions as it ranges from 0–100% and cannot represent incidence expressed by the number of people at risk of hospitalization. Defining incidence through routine data, affected by access and use of services, is complex [54] and the relationships between facility based TPR and incidence [55] and community prevalence [56] are non-linear. Nevertheless, routine surveillance of the public health impact of vector control will provide early warnings of a changing disease epidemiology. There is a growing body of evidence that suggests that the impact of IRS can be monitored through health facility and hospital data from routine health information systems in Uganda [13–15, 43, 56–62]. The continued surveillance of the health impact of IRS in Tororo is critical to understand possible further changes in TPR and the age-patterns of malaria admission.

## Conclusions

The findings from this study have important policy implications regarding malaria control in Uganda. The combination of IRS with LLIN distribution has a larger impact than LLIN alone. Sustained vector control increases the mean age of febrile test-positive admissions to hospital a likely result of delayed immune acquisition. However, even after many years of IRS the majority of febrile test positive are still among young children. LLINs and IRS used in combination will not be enough to achieve elimination in Uganda. Combinations of interventions that target vectors and the parasite might be required to interrupt transmission including MDA and vaccines. Routine enhanced health facility-based malaria surveillance data offers a cost-effective means of monitoring the impact of malaria control interventions on malaria infection rates over-time. Expanding the adoption of a standardized MRF for paediatric admissions across the country by the Ministry of Health, and regular interrogation of hospital data will provide insights into intervention impacts, failures and an epidemiological understanding of the changing clinical patterns of disease.

## Supplementary information

**Additional file 1: Table S1.** Summary of key indicators among hospitalized children at Tororo hospital: 2012 to 2019.

## Data Availability

The datasets used and/or analysed during the current study are available from the corresponding author on reasonable request.

## Abbreviations

AFI: Acute febrile illness
AL: Artemether-Lumefanthrine
IDI: Infectious Diseases Institute
IQR: Inter-quartile range
IRS: Indoor residual spraying
IQR: Inter quartile range
ITS: Interrupted time series
LLIN: Long-lasting insecticide-treated nets
MDA: Mass drug administration
MRF: Medical record form
MOH: Ministry of health
NMCP: National Malaria Control Programme
PMI: Presidents Malaria Initiative
RDT: Rapid Diagnostic Test
SD: Standard deviation
TPR: Test positivity rate
UMSP: Uganda malaria surveillance project
