# Supplementary material for: Changing malaria fever test positivity among paediatric admissions to Tororo district hospital, Uganda 2012–2019
Source: Malar J. 2020 Nov 19;19:416. doi: 10.1186/s12936-020-03490-4 (PMC7678291; doi:10.1186/s12936-020-03490-4)
Supplement: Supplementary file 1 — Additional file 1: Table S1. Summary of key indicators among hospitalized children at Tororo hospital: 2012 to 2019. [file 12936_2020_3490_MOESM1_ESM.docx]

Additional file 1: Table S1. Summary of key indicators among hospitalized children at Tororo hospital: 2012 to 2019

| **Variables** |  | **Year** | | | | | | | |
| --- | --- | --- | --- | --- | --- | --- | --- | --- | --- |
|  |  | **2012** | **2013** | **2014** | **2015** | **2016** | **2017** | **2018** | **2019** |
| Total admissions | N | 4829 | 5943 | 4217 | 3564 | 2267 | 3418 | 4019 | 3639 |
| Admissions with fever | n/N | 4516/4807 | 5512/5925 | 3681/3920 | 2937/3483 | 1801/2140 | 2935/3358 | 3503/4008 | 3164/3522 |
|  | (%) | (93.9%) | (93.0%) | (93.9%) | (84.3%) | (84.1%) | (87.4%) | (87.4%) | (89.8%) |
| Admissions with fever tested for malaria | n/N | 4406/4516 | 5385/5512 | 3301/3681 | 2869/2937 | 1288/1801 | 2571/2935 | 3448/3503 | 2616/3164 |
|  | (%) | (97.6%) | (97.7%) | (89.7%) | (97.7%) | (71.5%) | (87.6%) | (98.4%) | (82.7%) |
| TPR among all children tested with fever | n/N | 2578/4406 | 3340/5385 | 2264/3301 | 1176/2869 | 620/1288 | 1145/2571 | 661/3448 | 742/2616 |
|  | (%) | (58.5%) | (62.0%) | (68.6%) | (41.0%) | (48.2%) | (44.5%) | (19.1%) | (28.3%) |
| TPR among children (1-11 months) with fever | n/N | 812/1514 | 923/1646 | 551/925 | 249/793 | 111/343 | 140/553 | 78/883 | 90/585 |
|  | (%) | (53.6%) | (56.1%) | (59.6%) | (31.4%) | (32.3%) | (25.3%) | (8.8%) | (15.4%) |
| Age (months) for fever positives | Median (IQR) | 16 (9, 25) | 18 (10, 31) | 19 (12, 36) | 22 (12, 36) | 24 (13, 41) | 26 (17, 48) | 34 (18, 60) | 36 (18, 60) |
|  | Mean (SD) | 22 (20.7) | 25.3 (23.0) | 27.1 (24.6) | 30.1 (26.2) | 31.7 (26.6) | 35.8 (27.7) | 42.7 (33.0) | 46.0 (35.5) |
